# Sustained phospholipase C stimulation of H9c2 cardiomyoblasts by vasopressin induces an increase in CDP-diacylglycerol synthase 1 (CDS1) through protein kinase C and cFos

**DOI:** 10.1016/j.bbalip.2019.03.002

**Published:** 2019-07

**Authors:** Nicholas J. Blunsom, Evelyn Gomez-Espinosa, Tim G. Ashlin, Shamshad Cockcroft

**Affiliations:** Dept. of Neuroscience, Physiology and Pharmacology, Division of Biosciences, University College London, London WC1E 6JJ, UK

**Keywords:** PLC, phospholipase C, PIP_2_, phosphatidylinositol (4,5) bisphosphate, VP, Arg^8^-vasopressin, IP_3_, inositol (1,4,5) trisphosphate, GPCR, G-protein-coupled receptor, GRK, G-protein-coupled receptor kinase, CDS, CDP-diacylglycerol synthase, PA, phosphatidic acid, PI, phosphatidylinositol, PC, phosphatidylcholine, PGC-1α, peroxisome proliferator-activated receptor γ coactivator 1α, CDP-DG, CDP-diacylglycerol, DG, diacylglycerol, FCS, foetal calf serum, ER, endoplasmic reticulum, PIS, PI synthase, GRP75, 75 kDa glucose-regulated protein, PKC, protein kinase C, BIM-1, bisindolylmaleimide I, CHOP, the transcription factor CCAAT-enhancer-binding protein (C/EBP) homologous protein, IPs, inositol phosphates, PMA, Phorbol 12-Myristate 13-Acetate, Tg, thapsigargin, ERR, estrogen-related receptor, Protein kinase C, Phospholipase C, cFos, Phosphatidylinositol, Cardiac hypertrophy, Endoplasmic reticulum

## Abstract

Chronic stimulation (24 h) with vasopressin leads to hypertrophy in H9c2 cardiomyoblasts and this is accompanied by continuous activation of phospholipase C. Consequently, vasopressin stimulation leads to a depletion of phosphatidylinositol levels. The substrate for phospholipase C is phosphatidylinositol (4, 5) bisphosphate (PIP_2_) and resynthesis of phosphatidylinositol and its subsequent phosphorylation maintains the supply of PIP_2_. The resynthesis of PI requires the conversion of phosphatidic acid to CDP-diacylglycerol catalysed by CDP-diacylglycerol synthase (CDS) enzymes. To examine whether the resynthesis of PI is regulated by vasopressin stimulation, we focussed on the CDS enzymes. Three CDS enzymes are present in mammalian cells: CDS1 and CDS2 are integral membrane proteins localised at the endoplasmic reticulum and TAMM41 is a peripheral protein localised in the mitochondria. Vasopressin selectively stimulates an increase CDS1 mRNA that is dependent on protein kinase C, and can be inhibited by the AP-1 inhibitor, T-5224. Vasopressin also stimulates an increase in cFos protein which is inhibited by a protein kinase C inhibitor. We conclude that vasopressin stimulates CDS1 mRNA through phospholipase C, protein kinase C and cFos and provides a potential mechanism for maintenance of phosphatidylinositol levels during long-term phospholipase C signalling.

## Highlights

• Vasopressin stimulates phospholipase C activity over a 24 hour period leading to cardiac hypertrophy.
• Vasopressin causes a depletion in phosphatidylinositol levels.
• Vasopressin causes an increase in CDP diacylglycerol synthase 1 (CDS1) mRNA which is due to activation of protein kinase C.
• Protein kinase C activates cFos.
• cFos regulates the increase in CDS1 mRNA.

## Graphical abstract

**Figure:**
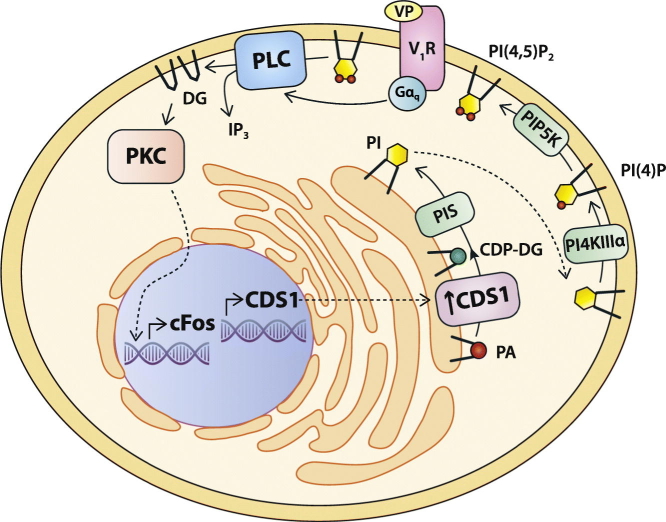

## 1 Introduction

Several agonists including norepinephrine, angiotensin II and vasopressin (VP) acting on Gq-protein coupled receptors induce hypertrophy of cardiac myocytes [[1], [2], [3], [4], [5]]. Gq-protein coupled receptors activate phospholipase C (PLC) resulting in the generation of the soluble second messenger, inositol (1,4,5) trisphosphate (IP_3_) and the membrane-bound diacylglycerol (DG). IP_3_ mobilises Ca^2+^ from intracellular stores of the endoplasmic reticulum (ER) whilst DG in the presence of Ca^2+^ activates protein kinase C (PKC). In addition to the generation of the second messengers, PLC activation can also result in the decrease in phosphatidylinositol (4,5) bisphosphate (PIP_2_) levels. PIP_2_ can regulate many cellular functions including the actin, cytoskeleton, ion channels and endocytosis. Besides Gq-protein-dependent PLC signalling, some receptors including the VP V_1_A receptor can also signal via G-protein independent, G-protein-coupled receptor kinase (GRK)-dependent mechanisms [6]. Ligand binding results in GRK-mediated phosphorylation of the C-terminal of the GPCR. β-Arrestins can bind to the phosphorylated C-terminal to act as a scaffold for additional signalling pathways.

Most studies focussing on the impact of VP signalling have been performed in neonatal cardiomyocytes and H9c2 rat myoblasts, where each express high levels of V_1_R [1,3,5]. The hypertrophic response to VP requires its continual presence for a period of 16–24 h. VP stimulates protein synthesis without an increase in cell number [1]. Cardiomyocyte cell lines such as H9c2 cells are established models for studying hypertrophy and show similar hypertrophic responses to primary neonatal cardiomyocytes in vitro [2]. The H9c2 cells are derived from embryonic rat ventricular myocytes and can be differentiated towards a more mature form by incubating the cells with all-*trans* retinoic acid in the absence of serum [[7], [8], [9]]. Differentiation promotes an increase in mitochondrial mass but the hypertrophic response is observed in both the undifferentiated and differentiated H9c2 cells [1,10].

**Fig. 1:**
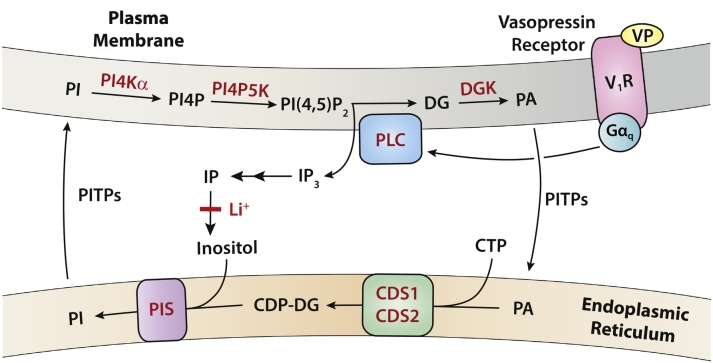
The PIP_2_ cycle. Stimulation of phospholipase C by VP acting on its G-protein-coupled receptor V_1_A results in the hydrolysis of PIP_2_ and formation of the second messengers, IP_3_ and DG. DG is phosphorylated to PA at the plasma membrane by DG kinase (DGK) and transferred to the ER. At the ER, PA and CTP are enzymatically converted to CDP-DG by CDS enzymes (CDS1 and CDS2). CDP-DG is synthesised into PI and this is catalysed by the enzyme PI synthase (PIS). PI is transferred to the plasma membrane for phosphorylation to PI(4,5)P_2_ by the resident enzymes, PI4KIIIα and PIP5K. Transfer of PI and PA transfer is carried out by members of the PITP family. Li^+^ blocks the activity of inositol monophosphatase thus allowing the accumulation of IPs. PI, phosphatidylinositol; PITPs, phosphatidylinositol transfer proteins; PI4P, PI 4-phosphate; PLC, phospholipase C; DG, diacylglycerol; PA, phosphatidic acid; IP, inositol phosphate, CDS, CDP-DG synthase; PIS, PI synthase; VP, vasopressin.

Phospholipase C (PLC) activation is accompanied by the resynthesis of PI via a series of enzymatic reactions at the ER, known at the ‘PIP_2_ cycle’ (see Fig. 1) [11,12]. Diacylglycerol (DG) is converted to phosphatidic acid (PA) at the plasma membrane by DG kinases and transported to the ER where it is converted into PI by two enzymes, CDP-diacylglycerol synthase (CDS) and PI synthase (PIS). The newly-synthesised PI is transported back to the plasma membrane via lipid transporters of the PITP family where it can be sequentially phosphorylated by the resident PI-4-kinase and PIP-5-kinase to PIP_2_ [11,13]. Reciprocal coupled transport of PA and PI is carried out by PITPNM1/RdgBα/Nir2 proteins [[14], [15], [16], [17]]. The rate-limiting step in the synthesis of PI is the CDS enzymes which catalyse the conversion of PA and CTP to CDP-DG. CDP-DG is essential for both PI and cardiolipin synthesis. There are three CDS enzymes in mammalian cells, which belong to two evolutionary distinct families (see Fig. 6A). TAMM41 is a peripheral membrane protein found exclusively on the inner mitochondrial membrane where it provides the substrate CDP-DG for cardiolipin synthesis [9,18]. In comparison, CDS1 and CDS2 are integral membrane enzymes localised to the ER; they show 73% identity and 92% similarity in their amino acid sequence but may exhibit very different expression patterns [19]. CDS2 is ubiquitously expressed whilst CDS1 is mainly expressed in brain, kidney and testis [19]. More recent analysis of mRNA levels indicates that CDS1 and CDS2 are expressed in most tissues (www.genecards.org). CDS1 and CDS2 enzymes show apparent selectivity for the different acyl chains of PA when examined in vitro using over-expressed enzymes [20]. In this study, CDS2 was found to prefer *sn*-1-stearoyl-*sn*-2-arachidonyl-PA as substrate whilst CDS1 showed no particular substrate specificity. In contrast, CDS1 was found to prefer *sn*-1-stearoyl-*sn*-2-arachidonoyl-PA as substrate although both PA from egg yolk lecithin and di-oleoyl-PA were also used [21]. PI species are unusual amongst the major class of lipids in that they are often found to have a highly restricted range of acyl chains with a C18:0 (stearoyl) chain at the *sn*-1 position and C20:4 (arachidonoyl) chain at the *sn*-2 position predominantly [22,23]. This is particularly the case for mammalian tissue as opposed to cultured cell-lines [22]. However, PI, once synthesised, undergoes acyl chain remodelling to acquire its final acyl chain specificity [[23], [24], [25], [26]].

In this study, we have examined how H9c2 cells maintain their PI levels during continuous stimulation of PLC when exposed to VP for extensive periods of time. We confirm that H9c2 cells show a hypertrophic response when exposed to VP for 24 h and demonstrate that this is accompanied by continuous stimulation of PLC activity. Phosphatidylinositol levels decreased and the cells compensate by increasing CDS1 mRNA. We identify protein kinase C and cFos to be responsible for the increase in CDS1 mRNA.

## 2 Material and methods

### 2.1 Materials

[Arg^8^]-VP (Cat. No. V9879) was purchased from Sigma-Aldrich. The AP-1 inhibitor, T-5224, was purchased from Cambridge Biosciences. GRP75 (mortalin) cloneN52a/42mouse monoclonal was from BioLegend. cFos antibody (Cat. No. #4384), p-AKT (s473; Cat. No. #9271), p-p42/44 MAPK (T202/Y204; Cat. No. #4370) and AKT (Cat. No. #9272) were obtained from Cell Signalling Technology. CHOP antibody (MA1250) was purchased from Life Technologies. GAPDH (MA5-15738) was from Thermofisher. Antibodies to PITPα PAb 674 were made in-house and have been described previously [27]. ZFPL1 [28] was a gift from Martin Lowe from the University of Manchester. Folch extract (brain extract Type 1 enriched in phosphoinositides was obtained from Sigma (Cat. No. B1502). Myo-[2-^3^H(N)]-inositol (Cat. No. NET114A005MC) was purchased from Perkin Elmer. Bisindolylmaleimide I (Cat. No. 203290) was purchased from Calbiochem.

### 2.2 Cell culture

H9c2 cells were cultured in Dulbecco's Modified Eagle Medium (DMEM; Invitrogen, 31966-021) supplemented with 10% heat-inactivated foetal calf serum (FCS) (Gibco, 10500-064), 0.5 iu.ml^−1^ penicillin and 50 μg.ml^−1^ streptomycin (Invitrogen, 15070-063), and grown in the incubator with 10% CO_2_ at 37 °C. For all experiments, except where indicated, the cells were transferred into M199 medium supplemented with 0.5 iu.ml^−1^ penicillin, 50 μg.ml^−1^ streptomycin and dialysed 1% FCS for 24 h. Incubation with VP was also carried out in the same medium. For experiments which required labelling with [^3^H]-inositol, the cells were also labelled in the same media. Medium199 was selected for labelling purposes as it contains 0.05 mg/l (0.28 μM) of myo-inositol compared to DMEM that contains 7.2 mg.l^−1^ (40 μM). Knock-down of CDS1 and CDS2 was carried out as described previously [9].

### 2.3 Immunofluorescence

H9c2 cells were grown on glass coverslips (19 mm, thickness 0). The cells were serum starved overnight and finally stimulated with 1 μM VP for 24 h in DMEM containing antibiotics, but no FCS. Cells were washed in phosphate-buffered saline (PBS) and subsequently fixed with 4% paraformaldehyde in PBS for 30 min at room temperature. Following a further two washes in PBS supplemented with 100 mM glycine, cells were permeabilised with PBS containing 0.2% Triton X-100 and 100 mM glycine for 10 min at room temperature. Cells were then washed again three times, before blocking with blocking buffer (0.1% BSA in PBS with 100 mM glycine), for 30 min at room temperature.

Cells were incubated with primary antibodies (GRP75 1:200 (mouse), ZFPL1 1:200 (sheep)) for 60 min at room temperature. The cells were washed 3 times for 3 min with the blocking solution. The specific secondary antibodies were then added, alongside the chemical stains; 49,6-diamidino-2-phenylindole (DAPI) (400 μg/ml) for nuclei and Rhodamine-phalloidin (7 nM) for actin, for 30 min at room temperature in the dark, diluted in the blocking buffer. Following incubation with the chemical stains and secondary antibodies, cells were washed three times with blocking buffer, three times with PBS, and finally rinsed with distilled water before mounting on a microscope slide with Fluoroshield (Sigma, F6182) and sealing the slide with nail varnish.

Quantification of cell length was monitored using CellSens Dimension Imaging software by Olympus.

### 2.4 Measurement of phospholipase C activity

Phospholipase C activity was monitored by measuring the release of labelled inositol phosphates (IPs) from H9c2 cells prelabelled with [^3^H]-inositol for 72 h. H9c2 cells were seeded confluently (~3.5 × 10^5^) and labelled for 3 days with [^3^H]-inositol (1 μCi/ml) in M199 supplemented with 1% dialysed FCS and antibiotics in 6 well plates. At the end of the third day, the cells underwent one of a variety of treatments: 24 h ± 1 μM VP in the presence of 10 mM LiCl, 24 h ± 1 μM VP with 10 mM LiCl only present for the final 20 min, and 24 h ± 1 μM VP with no LiCl added. In some experiments, the cells were stimulated for 20 min with ±1 μM VP and 10 mM LiCl.

Cells were stimulated at 37 °C in 10% CO_2_ for the indicated times. At the end of the incubation, the cells were placed on ice, the medium was removed and the cells quenched with 500 μl of ice-cold methanol. The cells were scraped and transferred to a chloroform-resistant tube. The wells were rinsed with a further 500 μl of methanol and combined with the first extract. Finally, the wells were washed with 900 μl water, which was added to the methanol extracts. Chloroform (1 ml) was then added directly to the tubes and the combined extracts were vortexed for 1 min. Following centrifugation to separate the phases, the IPs were recovered in the aqueous phase and were analysed on Dowex columns [29]. The [^3^H]-IPs were separated from inositol and glycerophosphoinositol by passage through Dowex 1 × 8 anion exchange resin. 1 ml of the aqueous extract was loaded on to columns. The columns were washed with 6 ml of water to remove inositol and glycerophosphoinositol was removed with 6 ml of 60 mM sodium tetraborate/5 mM sodium formate. [^3^H]-IPs were then eluted with 3 ml 1 M ammonium formate in 0.1 M formic acid directly into scintillation vials. The radioactivity was counted after addition of 5 ml Ultima-flo AF (Packard; Cat. No. 6013589).

The organic phase (containing the inositol-labelled phospholipids) had 1 ml of acidified synthetic top phase added to it, including 125 μg Folch extract (as a carrier). The samples were vortexed again thoroughly, and the organic layer was dried down overnight in a speedvac. The lipids were separated by TLC with the solvent composition chloroform (40): methanol (13): acetic acid (12): acetone (15): water (7). The TLC plates were phosphorimaged and analysed using AIDA software.

### 2.5 Measurement of phosphatidylinositol resynthesis

For the measurement of PI resynthesis, H9c2 cells were starved overnight and incubated in a HEPES buffer (20 mM HEPES, 137 mM NaCl, 3.7 mM KCl, 2 mM MgCl_2_, 1 mM CaCl_2_, 1 mg/ml BSA and 5.6 M glucose) containing 5 μCi of [^3^H]-inositol at 37 °C for 30 min. VP (1 μM) was added to the cells and incubated for a further 60 min. At the end of the incubation, medium was discarded and the lipids extracted as above and analysed by TLC using the solvent composition chloroform (75), methanol (45) acetic acid (3) and water (1) [30]. The TLC plate was exposed to iodine vapour to locate the lipids. The spot containing PI was scraped into scintillation vials and counted.

### 2.6 RNA isolation and real time PCR

H9c2 cells were seeded in 10 cm dishes. When cells were settled the media was changed to M199 + 1% dialysed FCS and the cells allowed becoming quiescent overnight. In the morning, cells had the agonist or inhibitor in the desired mix added to them: 1 μM VP, 5 μM BIM-I, 100 nM PMA, 0.5 μM Tg or 10 μM T-5224. Cells were left to incubate for 24 h unless stated otherwise. After stimulation, the cells were trypsinised, washed and transferred to Eppendorf tubes for total RNA extraction using the RNeasy Plus Mini Kit (Qiagen, Cat. No. 74134). RNA (1–5 μg) was reverse transcribed into cDNA using SuperScript™ II Reverse Transcriptase and random hexamer primers (Invitrogen). cDNA was diluted, and 313 ng of cDNA was loaded into each well of the PCR plate. Real-time quantitative PCR analysis was performed using KAPA SYBR® FAST qPCR kit Master Mix (KAPABIOSYSTEMS) and primers. (Specific *Rattus norvegicus* primers were designed using the website Primer 3 based on the NCBI sequences - available on request.) Quantitative PCR was performed using the CFX96 instrument (BioRad) and transcript levels were determined using the 2^−ΔΔCt^ method and normalized to PGK1 transcript levels [31].

### 2.7 CDS activity in control and vasopressin-stimulated membranes

H9c2 cells were seeded at 1.1 × 10^6^ cells per T175 flask, with 2 flasks per condition. Once the cells were confluent (~72 h), the media was replaced with DMEM supplemented with antibiotics, but without any FCS for 24 h. The cells were stimulated with 1 μM VP for 24 h. The cells were harvested, and the cell pellet resuspended in 0.2 M sodium bicarbonate (pH 11) to remove the peripheral protein, TAMM41, the CDS activity present in mitochondria. The bicarbonate buffer contained 1:100 dilution v/v protease inhibitor cocktail (Sigma, P8340). The cells were sonicated, and incubated at 4 °C on a rotating wheel for 60 min. After incubation, the membranes were recovered by centrifugation at 112,000*g* for 1 h at 4 °C. The pellet was resuspended in CDS buffer (50 mM Tris-HCL (pH 8.0), 50 mM KCl, 0.2 mM EGTA (ethylene glycol tetraacetic acid)) supplemented with 1/100 v/v protease inhibitor cocktail and sonicated once more. CDP diacylglycerol synthase (CDS) activity was determined exactly as described previously [9].

### 2.8 Western blotting

H9c2 cell were stimulated with VP and at the end of the incubation, the media was removed and the cells harvested in RIPA buffer with 1/100 v/v protease inhibitors. The protein content of the lysates was determined using the BCA (bicinchoninic acid) assay and the proteins (50 μg) were separated by SDS PAGE on Invitrogen NuPAGE 4–12% Bis-Tris gels. For Western blot, antibodies were used at the following dilutions: CHOP 1:1000; PITPα 1:1000; p-AKT (s473) 1:1000; AKT 1:1000; GAPDH 1:2500; p-p42/44 MAPK (T202/Y204) 1:1000; cFos 1:500.

### 2.9 Statistical analysis

Statistical analysis was performed using Prism 6 (GraphPad software for Science, San Diego, CA USA).

## 3 Results

### 3.1 Hypertrophic response of H9c2 cells with vasopressin treatment

**Fig. 2:**
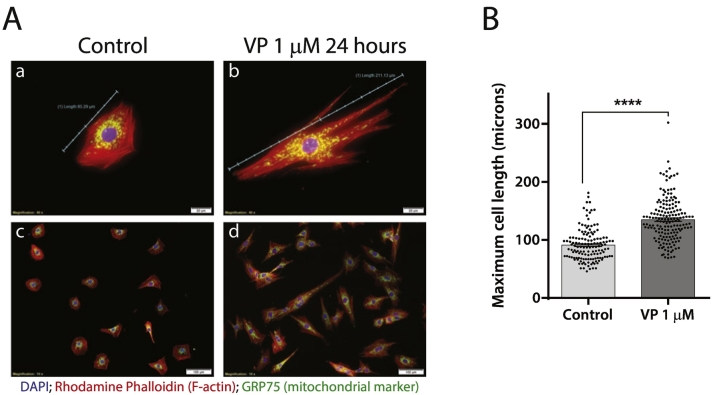
Vasopressin causes a hypertrophic phenotype in H9C2 cells. [A] H9c2 cells were cultured in DMEM without FCS overnight prior to addition of VP (1 μM) to the same medium for 24 h. The cells were fixed and stained with Rhodamine-phalloidin (F-actin), DAPI (nucleus) and GRP75 (mitochondrial marker). (a) The cell length of a control cell, 85.3 μm; (b) VP-stimulated cell, 211 μm. (a) and (b) at 40× magnification; (c) and (d) at 10× magnification. Scale bar in (a) and (b), 20 μm; and in (c) and (d), 100 μm. [B] The average cell length was measured and was found to increase from 91 to 135 μm. Unpaired two-tailed test P < 0.0001. Control, n = 140 cells; VP, n = 172 cells.

We initially confirmed that stimulation of H9c2 cells with VP causes hypertrophy. After transferring the cells in media containing 1% FCS overnight to render them quiescent, VP was added for 24 h. A characteristic hypertrophic response was observed in the morphology of the VP-stimulated cells (Fig. 2A); this includes an increase in cell size and increased protein synthesis. Enlargement of the cells was quantified by measuring the length of the cells (Fig. 2B). Additionally the number of cells in the culture dish were the same although the recovery of protein mass was ~23% higher (average of 13 experiments) indicating an increase in cell size.

### 3.2 Acute addition of vasopressin stimulates the phosphoinositide cycle

**Fig. 3:**
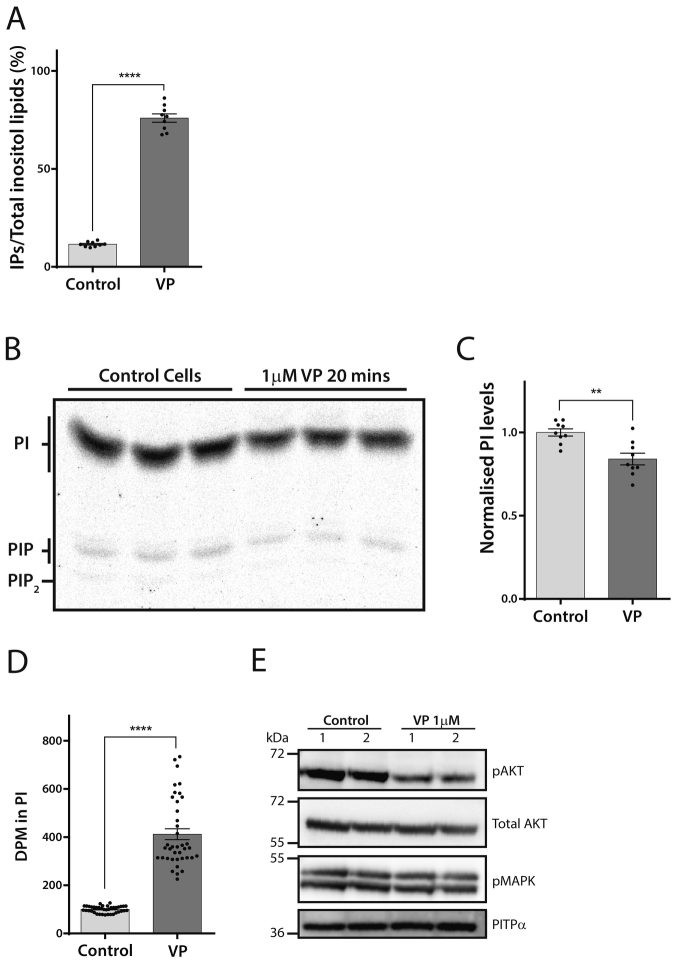
Acute addition of Vasopressin stimulates the PIP_2_ cycle in H9c2 cells. [A] H9c2 cells were labelled with [^3^H]-inositol for 48 h in M199 supplemented with 1% dialysed serum. VP (1 μM) was added for 20 min and the labelled IPs were monitored as an index of PLC activity. The IPs formed are expressed as a percentage of the total labelled inositol lipids. The results are from three independent experiments carried out in triplicate. [B] [^3^H]-inositol-labelled cells (as above) were stimulated with VP and the lipids extracted and separated by TLC. VP causes a reduction in phosphoinositide levels including phosphatidylinositol after 20 min of activation. The position of the different inositol lipids is indicated. [C] The decrease in PI was quantitated from three independent experiments carried out in triplicate. [D] VP stimulates the resynthesis of PI. H9c2 cells were pre-incubated with [^3^H]-inositol for 30 min after which buffer (control) or VP (1 μM) was added. After 60 min, the cell lipids were extracted for analysis of the incorporation of [^3^H]-inositol into PI. The results are from seven independent experiments carried out in triplicate. [E] Effect of VP on p-AKT (Ser^473^) and p-MAPK in H9c2 cells. VP causes a decrease in p-AKT and no effect on p-MAPK. Total AKT and PITPα were used as loading controls.

We first examined the signalling pathways activated by acute addition of VP. Addition of VP for 20 min results in the robust activation of PLC monitored by measuring the accumulation of [^3^H]-IPs in lithium chloride-treated cells. (Li^+^ inhibits the enzyme, inositol monophosphatase) (see Fig. 1, Fig. 3A). (Confluent H9c2 cells were prelabelled by maintaining the cells in the presence of [^3^H]-inositol for 72 h). Although the substrate for PLC is mainly PI(4,5)P_2_, it is rapidly regenerated by sequential phosphorylation of PI (Fig. 1). PI is the main phosphoinositide of the total pool of phosphoinositides and thus the amount of labelled IPs formed during stimulation normally exceeds the amount of labelled PIP_2_ present at the start [29]. Stimulation with VP for 20 min led to a reduction in all three phosphoinositides, PI, PIP and PIP_2_ (Fig. 3B). The PI levels decreased by ~20% (Fig. 3B and C). Importantly, compensatory resynthesis of PI (see pathway in Fig. 1) was also observed (Fig. 3D). This was monitored by incubating the H9c2 cells with VP in the presence of [^3^H]-inositol for 1 h. A robust increase in PI labelling reflecting enhanced synthesis was observed (Fig. 3D).

We also monitored the effects of VP stimulation on AKT and MAP kinase phosphorylation. Addition of VP for 20 min caused a substantial reduction in AKT phosphorylation and no changes in MAP kinase phosphorylation (Fig. 3E).

### 3.3 Sustained phospholipase C activation of H9c2 cells with vasopressin

**Fig. 4:**
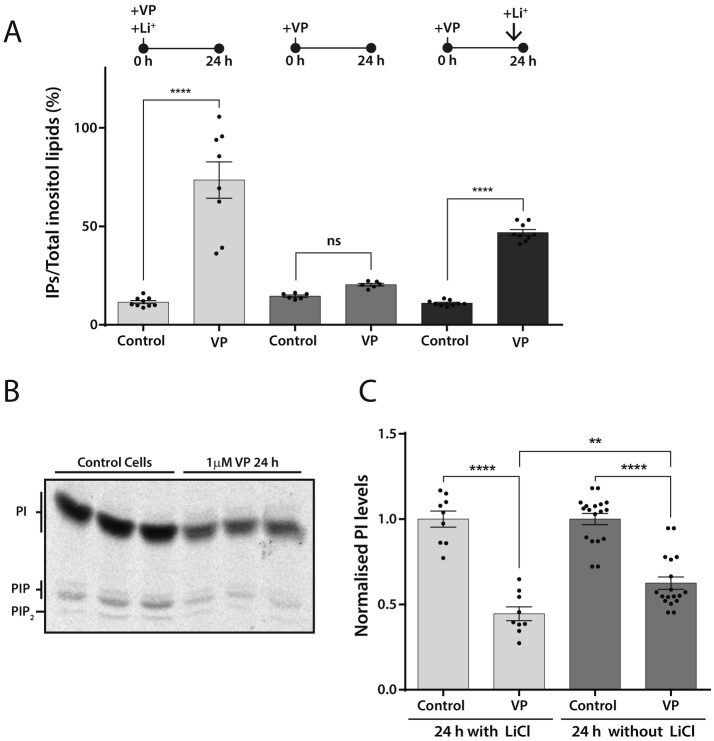
Sustained 24 h stimulation with vasopressin maintains continuous phospholipase C activity and is accompanied by a reduction in phosphatidylinositol levels. [A] PLC activity is maintained over a 24 h period. [^3^H]inositol-labelled cells were stimulated with VP for 24 h in the presence of Li^+^ (light grey bars), stimulated with VP in the absence of Li^+^ (grey bars), or stimulated with VP with Li^+^ added for the last 20 min of the incubation (dark grey bars). [B] [^3^H]-inositol-labelled cells were stimulated with VP *plus* Li^+^ for 24 h and the lipids extracted and separated by TLC. VP causes a strong reduction in phosphoinositides levels including PI after 24 h of stimulation. [C] Quantification of the decrease in PI levels after treatment with VP for 24 h in the presence or absence of LiCl. The presence of Li^+^ has only a small impact on the decrease in PI levels.

Phospholipase C plays an important role in the signal transduction mechanisms of cardiac hypertrophy. For hypertrophic responses, cardiomyocytes or H9c2 cells need to be exposed to VP for a sustained period e.g. 16–24 h (Fig. 2). Although VP stimulates PLC activity when acutely added (Fig. 3), it is not known whether the PLC activity is sustained for long periods. We first examined PLC activation after a 24 h period with VP by monitoring the accumulation of [^3^H]-IPs in the presence of Li^+^. An increase in IPs was observed that was entirely dependent on the presence of Li^+^ (Fig. 4A; compare the light grey bars with the grey bars). In order to examine whether PLC activity remains continuously active over a 24 h period, we stimulated the cells with VP for 23 h 40 min in the absence of Li^+^ (under these conditions no IPs would accumulate). Li^+^ was added for the final 20 min only (Fig. 4A, dark grey bars). A robust increase in [^3^H]-IPs is observed (Fig. 4A; dark grey bars). This result confirms that PLC remains continuously active even after 24 h of VP treatment.

The impact of PLC activity on the levels of PI was simultaneously monitored. Sustained stimulation over 24 h led to a large drop in PI levels (Fig. 4B). A drop in PIP and PIP_2_ was also evident. In principle, the presence of Li^+^ might affect the availability of inositol for the resynthesis pathway and therefore influence the resynthesis of PI. To examine this, we also monitored the decrease in PI levels after VP stimulation in the absence of Li^+^. The reduction in PI upon VP stimulation after 24 h was slightly less compared to that observed in the presence of Li^+^ (Fig. 4C).

**Fig. 5:**
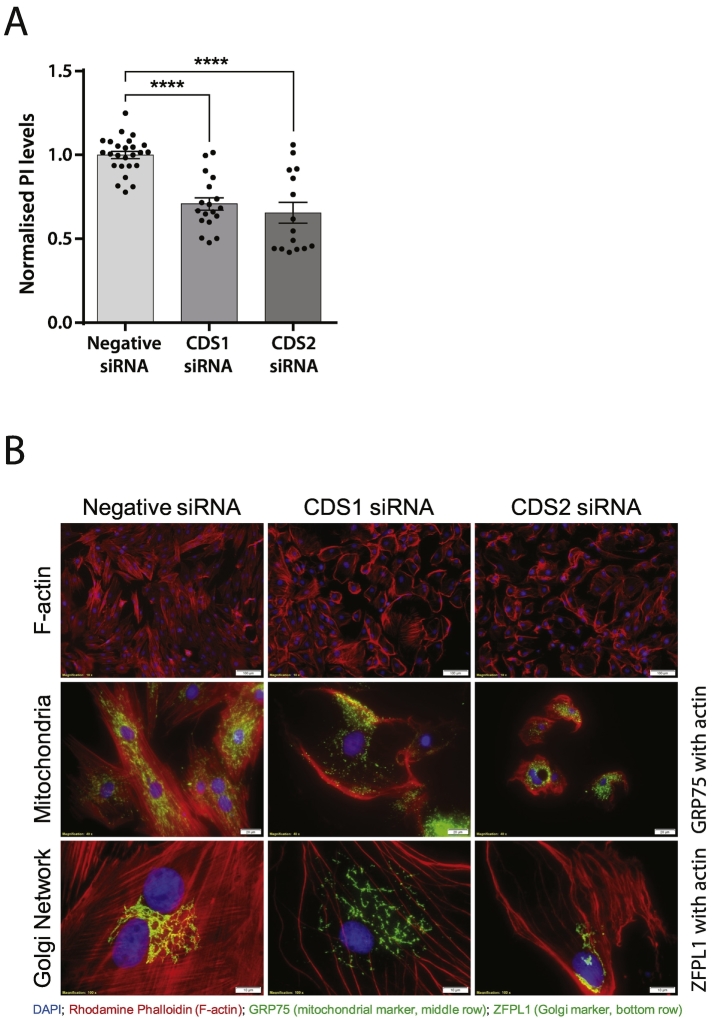
CDS1 and CDS2 knockdown decreases phosphatidylinositol levels and causes multiple morphological changes. [A] Knockdown of CDS1 or CDS2 causes a reduction in PI levels. H9c2 cells were treated with siRNA for CDS1 or CDS2 during the labelling of the cells with [^3^H]-inositol. [B] H9c2 cells were treated with siRNA for CDS1 or CDS2 and the cells were stained with Rhodamine Phalloidin (F-actin), GRP75 (mitochondrial marker) and ZFPL1 (Golgi marker). Knockdown or either CDS1 or CDS2 cause derangement of the actin cytoskeleton; and Golgi and mitochondrial fragmentation.

Previous studies have suggested that CDS2 is the enzyme required for the resynthesis of PI after PLC activation because of its preference for PA enriched in stearic and arachidonic acid (see Fig. 1) [20]. We used siRNA to silence CDS1 and CDS2 as described previously [9]. Knockdown of CDS1 or CDS2 caused a reduction in PI levels (Fig. 5A) as well as in PIP and PI(4,5)P_2_. Upon examination of the cells by microscopy, it was observed that the cells had significant morphological changes. In particular, the actin filaments were disrupted, and the Golgi and the mitochondrial network was fragmented (Fig. 5B). Recovery of the CDS knockdown cells after stimulation with VP was compromised making it difficult to test the requirement of CDS1 versus CDS2 during resynthesis of PI.

### 3.4 Vasopressin stimulates an increase in CDS1 mRNA

**Fig. 6:**
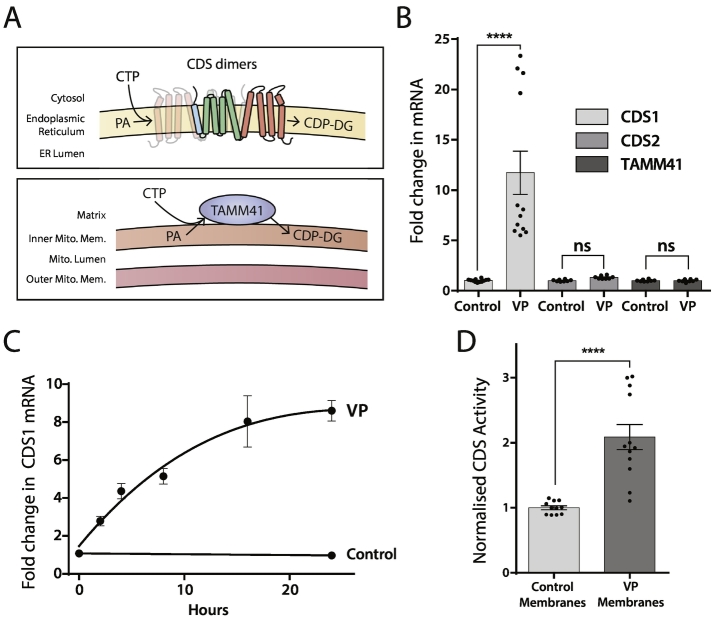
VP stimulates an increase in CDS1 mRNA and CDS activity in membranes. [A] CDS enzymes present in the mammalian genome. CDS1 and CDS2 are dimeric integral membrane proteins whilst TAMM41 is a peripheral inner mitochondrial protein. CDS proteins have three domains, the N-terminal domain (blue), the dimerisation interface (green) and a highly conserved C-terminal domain (red). [B] VP specifically increases CDS1 mRNA in H9c2 cells. H9c2 cells were stimulated for 24 h with VP. The cells were lysed and the mRNA for CDS1, CDS2 and TAMM41 monitored by PCR. Results are shown from three independent experiments. One way ANOVA using Sidak's multiple comparison test. P < 0.0.0001. CDS1, n = 12; CDS2, n = 8; TAMM41, n = 8. [C] Time-course of CDS1 mRNA stimulated with VP. [D] VP stimulates an increase in CDS activity in total membranes. H9c2 cells were stimulated with VP for 24 h. The cells were sonicated and the resulting membranes incubated with 0.2 M sodium bicarbonate buffer (pH 11) to remove the peripheral protein, TAMM41. The membranes were recovered after centrifugation and assayed for CDS activity. Unpaired two-tailed *t*-test P < 0.0001. Control, n = 11; AVP, n = 12.

The rate-limiting enzyme for PI resynthesis is CDS (Fig. 1). To examine whether sustained stimulation affects the expression of the CDS enzymes, we monitored mRNA levels of the three CDS enzymes. CDS1 and CDS2 are related enzymes expressed at the ER whilst the unrelated enzyme TAMM41 is present in the mitochondria [9] (Fig. 6A). VP present for 24 h stimulates an increase in the mRNA of CDS1 with no changes in CDS2 or TAMM41 (Fig. 6B). The increase in CDS1 mRNA ranged from 6 to 20 fold. The increase in CDS1 mRNA plateaued at about 16–24 h (Fig. 6C).

As no validated antibodies to CDS1 are available (see [9]), we monitored the CDS activity in membranes after stripping the peripheral proteins with sodium bicarbonate buffer to remove TAMM41 (Fig. 6D). An increase in CDS activity was observed in membranes prepared from VP-stimulated cells indicating that the increase in mRNA is likely accompanied by an increase in protein levels.

We also examined whether VP treatment affected any other components of the PIP_2_ resynthesis pathway, specifically, PIS and Nir2/PITPNM1. VP had no effect on either PIS or Nir2 mRNA.

### 3.5 The increase in CDS1 mRNA is dependent on protein kinase C

**Fig. 7:**
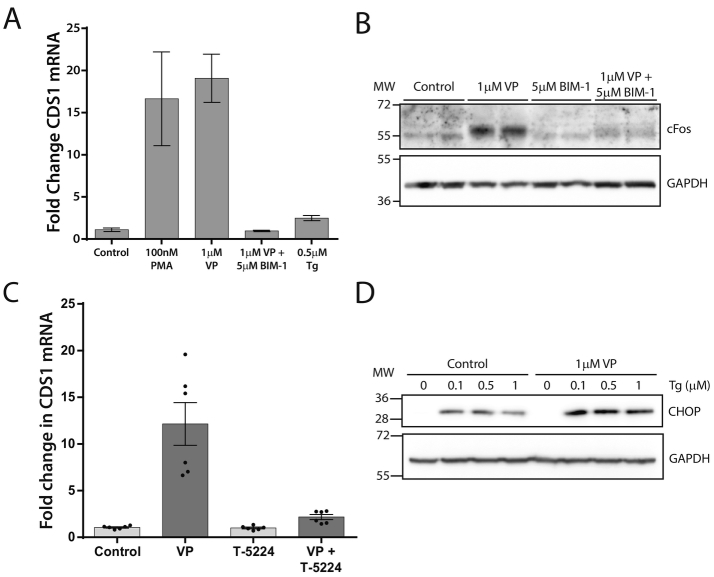
Activation of protein kinase C followed by a rise in cFos is required for the increase in CDS1 mRNA by vasopressin. [A] H9c2 cells were stimulated with VP (1 μM), PMA (100 nM) or thapsigargin (0.5 μM) for 24 h. BIM-1 (5 μM), protein kinase C inhibitor was added together with VP. CDS1 mRNA levels were monitored by real time PCR. [B] VP stimulates an increase in cFos, which is dependent on protein kinase C. GAPDH was used as a loading control. [C] VP-stimulated CDS1 mRNA is inhibited by the AP-1 inhibitor, T-5224 (10 μM). [D] Treatment of H9c2 cells with VP for 24 h does not cause a stress response but enhances the thapsigargin-stimulated stress response. H9c2 cells were stimulated with or without VP (1 μM) in the presence of thapsigargin (0.1, 0.5 and 1 μM) for 24 h. The samples were processed for Western blot. CHOP antibody: 1:1000; GAPDH 1:1000 was used loading control.

VP stimulation causes an increase in cytosol Ca^2+^ and in PKC activity. To examine whether the increase in CDS1 mRNA was dependent on PKC activity, we used PMA to directly activate PKC. PMA stimulates an increase in CDS1 mRNA similarly to VP (Fig. 7A). To confirm that PKC was responsible when VP was used as a stimulus, the PKC inhibitor, bisindolylmaleimide I (BIM-1) was applied. As shown in Fig. 7A, the increase in CDS1 mRNA by VP was inhibited by the PKC inhibitor, BIM-1. In contrast, thapsigargin treatment which can increase cytosol Ca^2+^ was without effect on CDS1 mRNA levels.

### 3.6 Vasopressin causes an increase in cFos

The protein cFos is a member of the AP-1 family of inducible transcription factors whose expression is tightly regulated. It has been previously shown that PKC is involved in PLC-mediated increases in cFos gene expression in adult cardiomyocytes [32,33]. We therefore examined whether VP stimulates an increase in cFos in H9c2 cells and whether this might be responsible for the increase in CDS1 mRNA. VP stimulates a robust increase in cFos (Fig. 7B). The increase in cFos is dependent on PKC as it is inhibited by the PKC inhibitor, BIM-1 (Fig. 7B).

To examine whether cFos is responsible for the increase in VP-stimulated CDS1 mRNA we took advantage of an AP-1 inhibitor, T-5224 [34,35]. AP-1 contains members of the Fos and Jun families, which form either Jun-Jun homodimers or Fos-Jun heterodimers and bind to the consensus DNA sequence 5′-TGAGTCA-3′, which is known as the AP-1 binding site. T-5224 inhibits the activity of the cFos/cJun AP-1 heterodimer [36,37]. The stimulated increase in CDS1 mRNA by VP was inhibited by T-5224 confirming that CDS1 mRNA is regulated by PKC-cFos pathway (Fig. 7C).

### 3.7 A decrease in PI levels in vasopressin-stimulated H9C2 cells does not cause ER stress

In zebrafish larvae, lack of de novo PI synthesis due to a mutation on PIS led to ER stress specifically in intestinal epithelial cells [38,39]. These cells showed disruption of cellular architecture, mitochondrial defects and increased autophagy and cell death. Such disruption was also noted in the H9c2 cells knocked down for CDS1 and CDS2 (Fig. 5B). These observations imply that loss of PI can cause ER stress. To examine whether the decrease in PI levels that occur with vasopressin stimulation causes ER stress, we monitored CHOP, a transcription factor expressed during ER stress [40]. Vasopressin failed to induce ER stress unlike thapsigargin which was found to increase CHOP protein. However, it was noted that the increase in CHOP by thapsigargin was enhanced when the cells had also been treated with VP (Fig. 7D).

## 4 Discussion

### 4.1 Vasopressin increases CDS1 mRNA via protein kinase C and cFos

The mammalian heart is a dynamic organ that enlarges in response to physiological and pathological stimuli due to the increase in size of individual cardiac myocytes. Multiple lines of evidence support the importance of the Gαq-phosphoinositide signalling system in the development of pathological hypertrophy [41,42]. Gαq-protein coupled receptor (GPCR) agonists such as angiotensin II, VP, endothelin-1, and phenylephrine activate PLC-mediated hydrolysis of PI(4,5)P_2_, which leads to the activation of PKC by DG and by a rise in cytosol Ca^2+^ by I(1,4,5)P_3_. Previous studies have demonstrated that PKC activation plays an important role in the development and progression of cardiac hypertrophy [43,44].

In this study, we have examined the impact of sustained VP signalling on phosphatidylinositol turnover during PLC signalling in a cardiac cell-line, H9c2. Under these conditions, H9c2 cells undergo hypertrophy. Our findings indicate that VP stimulates PLC activity continuously over the 24 h period and PI levels are substantially reduced. To compensate for this loss, PI resynthesis is increased. The rate limiting step in the resynthesis of PI is the enzyme CDS and here we show that of the two ER-localised CDS enzymes, CDS1 expression is increased following VP stimulation. Other studies in zebrafish and *Drosophila* also suggest that CDS activity regulates both the availability of PIP_2_ and the extent of PIP_2_-dependent signalling. In zebrafish, CDS-dependent phosphoinositide availability limits VEGF-A signalling [45]. Like humans and mice, zebrafish have two CDS genes, *cds1* and *cds2*. *Cds2* mutants result in vascular-specific defects in vivo and this is due to the failure of VEGF-A-stimulated PLC activity; the phenotype could be rescued by exogenous PIP_2_ indicating that CDS2 controlled the supply of PIP_2_. It is noteworthy that experiments done in vitro using HUVEC endothelial cells, found that knockdown of either CDS1 or CDS2 cause defective invasion and reduced ERK activation, an in vitro model for the vascular defects observed in the animals. *Drosophila* expresses a single *cds* gene, and a mutation in *cds* displays light-induced irreversible loss of phototransduction and retinal degeneration [46]. Phototransduction in flies is entirely dependent on PLC activation and it was reported that the amplitude of the light response in *Drosophila* photoreceptor cells is modulated by Cds levels, showing that Cds-dependent PIP_2_ recycling limits PLC–mediated phototransduction in the fly retina [[46], [47], [48]].

VP causes an increase in CDS1 but not CDS2 mRNA. CDS1 and CDS2 are integral membrane proteins and can form homodimers [9]. Likewise, the Cds enzyme from the bacterium *Thermotoga maritima* is also a dimer [49]. We note from Biogrid database that CDS1 and CDS2 also interact (https://thebiogrid.org/114295/table/homo-sapiens/cds2.html) suggesting that they may form heterodimers. Previous studies using different chain lengths of PA as substrate suggested that CDS2 has a preference for 1-stearoyl–2-arachidonoyl PA whilst CDS1 has no preference [20]. However, another study using CDS1 showed that CDS1 had a preference for 1-stearoyl-2-arachidonoyl PA [21]. The substrate specificity for the heterodimer is unknown. In cardiomyocytes, the predominant DG kinase is the zeta form and cardiac-specific over-expression in mice blocks the GPCR-agonist-induced activation of the DG-PKC signalling thus inhibiting cardiac hypertrophy [41]. Interestingly, DG kinase zeta appears to prefer C16:C16-DG as substrate [50]. Thus, it would appear that the PA formed during PLC signalling in cardiomyocytes might not possess the canonical fatty acid composition. It is noteworthy that the analysis of the fatty acid composition of PI from a variety of cultured cell lines and tissues indicate a variable amount of enrichment in stearoyl-arachidonoyl PI [22,51]. Further studies will be needed to firmly establish the fatty acid profiles of the intermediates of the PIP_2_ cycle in cardiomyocytes.

In H9c2 cells, activation of PKC is required for the increase in CDS1 mRNA as inhibitors of PKC block the response. Moreover, PMA increases CDS1 mRNA. In a previous study, PMA treatment for 18–24 h of C3A human hepatoma cells resulted in increased PI synthesis which was sensitive to inhibition by PKC inhibitors [52]. We suggest that this might be due increased expression of CDS1 mRNA as shown for H9c2 cells here. Other studies using cardiomyocytes have reported that norepinephrine stimulates cFos expression due to PLC activity via PKC [33]. cFos is a transcription factor and in VP-stimulated cells, cFos expression is also increased in a PKC-dependent manner. Whilst our work identifies cFos as a regulator of CDS1 mRNA expression, a previous study has shown that an increase in cFos protein causes an increase in phosphoinositide labelling and this was due to the direct activation of CDS1 by cFos at the ER [53,54]. A physical interaction between the N-terminal domain of cFos and CDS1 was found to increase CDS1 activity in vitro.

### 4.2 The CDS1 and CDS2 genes are highly regulated

The *CDS1* gene appears to be a highly regulated gene. Other studies have reported regulation by different mechanisms (summarised in Table 1). In PGC-1α/β heart-specific knockout mice, there is a decrease in *CDS1* expression but an increase in *CDS2* mRNA expression. Moreover, expression of PGC-1α or -1β increased *CDS1* mRNA in neonatal rat cardiac myocytes [55]. Estrogen-related receptor (ERR) is a well-characterised PGC-1α co-activator target [56] and two conserved ERR-binding site sequences present at the *CDS1* promoter region was found to be responsible for PGC-1α-dependent activation. Yet another regulator is ZEB1, an E-Box transcriptional repressor [57]. The expression of CDS1 mRNA is inversely correlated with ZEB1 in a series of 22 NSCLC (non-small cell lung cancer) cell-lines. This result was confirmed by over-expression of ZEB1 in H358 cells where a decrease in CDS1 mRNA was noted whilst knockdown of ZEB1 resulted in increased CDS1 mRNA expression. An increase in CDS1 but not CDS2 mRNA was also found during the differentiation of 3T3-L1 preadipocytes to adipocytes and knockdown of CDS1 inhibited adipocyte differentiation [58].

**Table 1:** Regulation of CDS1 and CDS2 mRNA by different mechanisms.

Treatment	Up-regulated	Down-regulated	Comments	References
ZEB1 over-expression		*CDS1*	Cells expressing ZEB1 at high levels correlated with low CDS1 levels.	[57]
PGC-1α/β heart-specific knockout mice	*CDS2*	*CDS1*	Gene expression profiling revealed reduced expression of CDS1	[55]
PGC-1α or PGC-1β over-expression	*CDS1*		Over-expression in Neonatal rat cardiac myocytes	[55]
Vasopressin	*CDS1*		H9c2 cells stimulated for 16 h. CDS1 expression inhibited by PKC and AP-1 inhibitor	This paper
Palmitic acid via p53 with SIRT6	*CDS1 and CDS2*		p53 and SIRT6 bind the promoters of CDS1 and CDS2	[60]
PMA	*CDS1*		H9c2 cells stimulated for 24 h.	This paper
Adipocyte differentiation	*CDS1*		3T3-L1-preadipocytes differentiated in vitro for 8 days	[58]

Palmitic acid, known to cause ER stress [59], also promotes an increase in both CDS1 and CDS2 mRNA [60]. Co-occupancy of p53 and SIRT6 on CDS1 and CDS2 promoters is responsible for increased gene expression [60]. It is noteworthy that sustained VP stimulation does not lead to ER stress unlike thapsigargin. Thapsigargin causes ER stress by depleting ER Ca^2+^ stores. Although IP_3_ would also deplete ER Ca^2+^ stores, these stores get replenished [61]. In H9c2 cells, ER stress induced by thapsigargin is not accompanied by an increase in CDS1 mRNA (Fig. 7).

### 4.3 Knockdown of CDS1 and CDS2 phenotypes

Knockdown of CDS1 and CDS2 has detrimental effects on the H9c2 cells not dissimilar to that observed in the intestinal epithelial cells of the zebrafish larvae [39]. The simplest explanation for this drastic phenotype is the loss of PI and its phosphorylated derivatives, PIP and PIP_2_, which have significant effects on both the Golgi and the actin cytoskeleton respectively. Thus, why are VP-stimulated cells protected from this fate as they also show a decrease in the phosphoinositide levels? Indeed, a higher drop in PI levels is recorded with VP compared to the cells knocked down for CDS1 or CDS2. The answer must lie in the ability of VP to stimulate PI resynthesis and the increased expression of CDS1 would assist in increasing the rate. Moreover, de novo PI synthesis would be unaffected in VP-stimulated cells. Thus in VP-stimulated cells, the on-going PI resynthesis must provide protection in contrast to the knockdown cells, where de novo PI synthesis would also be affected. The observation that knockdown of either CDS1 or CDS2 leads to loss of PI suggest that both enzymes contribute in maintaining PI levels. Similar observations have been reported for HeLa cells knocked down for either CDS1 or CDS2 [58].

### 4.4 Vasopressin causes a reduction in AKT phosphorylation

In addition to stimulating PLC, VP caused a decrease in the phosphorylation of AKT (Fig. 3E). Activation of AKT requires sequential phosphorylation by PDK1 at Ser^473^ and mTORC2 complex at Thr^308^, which is dependent on PI(3,4,5)P_3_ binding to the pleckstrin homology domain of AKT [62]. These phosphorylations are reversed by protein phosphatase 2A. Some GPCRs are rapidly phosphorylated by members of a family of GPCR kinases (GRKs) which in turn leads to the recruitment of β-arrestins. β-arrestin-2 can act as a scaffold for the formation of a signalling protein complex allowing GPCRs to signal independently of G-proteins. The formation of such a signalling complex comprising of p-AKT, β-arrestin-2, and protein phosphatase 2A has been described for D2 dopamine receptors, which leads dephosphorylation and inactivation of AKT in the presence of dopamine [63]. Another example is glutamate, which also causes a decrease in AKT phosphorylation in neurons [64]. VP receptors also appear to regulate AKT phosphorylation very likely by a similar mechanism. However, we cannot exclude the possibility that PIP_2_ depletion by PLC could reduce substrate availability for PIP_3_ production that could account for the decrease in AKT phosphorylation.

## 5 Conclusion

In conclusion, H9c2 cells respond to chronic VP stimulation by upregulating the CDS1 mRNA. The increase in CDS1 mRNA is dependent on protein kinase C which increases cFos. We suggest that the increase in CDS1 mRNA and consequently its activity would hasten the replenishment of the pool of PI and thus restore PIP_2_ levels.

## Conflict of interest

The authors declare that they have no conflicts of interest with the contents of this article.

## Transparency document

Transparency document. Image 1
